# Simultaneous MEG-LFP Recordings to Assess In Vivo Dystonic Neurophysiological Networks: A Feasibility Study

**DOI:** 10.3390/brainsci15121268

**Published:** 2025-11-26

**Authors:** Elisa Visani, Lorenzo Bergamini, Chiara Gorlini, Dunja Duran, Nico Golfrè Andreasi, Giovanna Zorzi, Eleonora Minacapilli, Davide Rossi Sebastiano, Paola Lanteri, Daniele Cazzato, Roberto Eleopra, Vincenzo Levi

**Affiliations:** 1 SC Epilettologia Clinica e Sperimentale, Fondazione IRCCS Istituto Neurologico Carlo Besta, Via Celoria 11, 20133 Milan, Italy; elisa.visani@istituto-besta.it (E.V.); dunja.duran@istituto-besta.it (D.D.); 2 SSD Neurochirurgia Funzionale, Fondazione IRCCS Istituto Neurologico Carlo Besta, Via Celoria 11, 20133 Milan, Italy; lorenzo.bergamini@istituto-besta.it (L.B.);; 3 SC Malattia di Parkinson e Disordini del Movimento, Fondazione IRCCS Istituto Neurologico Carlo Besta, Via Celoria 11, 20133 Milan, Italy; nico.golfreandreasi@istituto-besta.it (N.G.A.); roberto.eleopra@istituto-besta.it (R.E.); 4 SC Neuropsichiatria Infantile e Disordini del Movimento, Fondazione IRCCS Istituto Neurologico Carlo Besta, Via Celoria 11, 20133 Milan, Italy; giovanna.zorzi@istituto-besta.it (G.Z.); eleonora.minacapilli@unimi.it (E.M.); 5 SC Neurofisiopatologia, Fondazione IRCCS Istituto Neurologico Carlo Besta, Via Celoria 11, 20133 Milan, Italy; davide.rossi@istituto-besta.it (D.R.S.); paola.lanteri@istituto-besta.it (P.L.); daniele.cazzato@istituto-besta.it (D.C.)

**Keywords:** DBS, local field potentials (LFP), MEG, dystonia

## Abstract

**Background/Objectives:** Subcortical local field potentials (LFPs) provide a valuable in vivo window into the neurophysiology of the dystonia network. These signals can be recorded through Deep Brain Stimulation (DBS) devices and combined with whole-head techniques such as magnetoencephalography (MEG) to study cortical–subcortical interactions. However, simultaneous LFP-MEG acquisition poses challenges, including interference from the DBS device and synchronization issues. We present preliminary data on the feasibility and signal quality of concurrent LFP and MEG recordings in dystonia patients. **Methods:** We assessed simultaneous MEG-LFP recordings in 11 patients with inherited or idiopathic dystonia who underwent bilateral DBS lead implantation in the Globus Pallidus Internus (GPi). Two synchronization strategies were tested: (1) the Tapping method, using an accelerometer placed on the DBS device, and (2) the Stimulation method, which generated detectable artifacts during sham stimulation. **Results:** Both methods successfully aligned MEG and LFP signals with a mean temporal delay of 91 ± 22 ms for the Tapping method and 288 ± 166 ms for the Stimulation method. Post-implantation signal-to-noise ratio analysis revealed slight degradation but no significant impact on MEG quality (gradiometers: −0.12 ± 1.85 dB; magnetometers: −0.47 ± 2.03 dB). **Conclusions:** Simultaneous MEG-LFP recordings in dystonic patients are feasible, yielding high-quality signals, and reliable synchronization. Temporal alignment improved with practice, suggesting a short learning curve. This method opens new opportunities to study cortical-subcortical dynamics and strengthens the potential of combining MEG-LFP approaches for investigating dystonia.

## 1. Introduction

Advancements in neuroimaging and neural recording techniques have enabled the simultaneous acquisition of several neurophysiological signals, providing an integrated approach to a more comprehensive understanding of the whole-brain dynamics. One approach involves the concurrent measurement of local field potentials (LFPs) during deep brain stimulation (DBS) and neuromagnetic signals acquired through magnetoencephalography (MEG).

These two modalities provide complementary information. LFPs reflect the local extracellular electrical potentials recorded directly from subcortical structures [1]. MEG non-invasively measures the magnetic fields generated primarily by intracellular postsynaptic currents in synchronously active cortical pyramidal neurons [2]. While electroencephalography (EEG) is a widely used method for recording cortical activity, MEG offers distinct advantages in this context. Unlike the electrical potentials measured by EEG, which are attenuated by the variable conductivity of the skull and scalp, magnetic fields pass through these tissues with minimal distortion. This grants MEG superior spatial resolution and facilitates more precise localization of the signal sources compared to EEG, while maintaining the same temporal resolution [3].

Intracranial recordings have been extensively characterized, particularly in the context of epilepsy and Parkinson’s disease (PD). In PD, for instance, LFP recordings from the subthalamic nucleus (STN) have been crucial in identifying beta-band oscillations as a biomarker of motor impairment [4,5]. The information derived from LFPs provides high-precision local source dynamics, offering insights into neural synchrony and is conceptually comparable to source localization techniques or dipole modeling. However, while the electrophysiological signatures of PD are well-documented, the LFP characteristics in dystonia—typically targeted at the globus pallidus internus (GPi)—are less extensively studied and exhibit distinct spectral features, such as enhanced low-frequency pallidal activity [6,7].

The combination of LFP and MEG enables the investigation of cortical-subcortical dynamics by revealing oscillatory coherent networks and their frequency and topography [8,9]. The most challenging aspect of LFP-MEG recordings lies in the difficulty of acquiring both signals simultaneously, as they are typically recorded using independent and unsynchronized equipment. Integrating these techniques requires overcoming several technical hurdles, including signal synchronization across different systems, the management of noise and artifacts intrinsic to each recording method, and aligning data at the temporal level. Among all the issues described, the most significant challenge is aligning the signals. This alignment is crucial because it is not possible to physically couple the recording devices, as one is implanted (the DBS) and the other is external (the MEG).

We report the technical considerations, synchronization strategies and preliminary results of simultaneous MEG-LFP acquisition from globus pallidus internus (GPi) in a cohort of dystonic patients. Our results demonstrated the potential for future studies to leverage this combined approach for a more accurate understanding of cortico-subcortical interactions in dystonia is essential for elucidating the pathophysiology of the disorder and may contribute to the optimization of DBS stimulation.

## 2. Materials and Methods

### 2.1. Patients

This study included all the 11 patients who underwent stereotactic placement of bilateral leads in the GPi for inherited or idiopathic generalized drug-resistant dystonia at Fondazione IRCCS Istituto Neurologico Besta between December 2023 and February 2025. Individual demographic and clinical features for each patient are reported in Table 1. There were 8 patients with inherited generalized dystonia and 3 patients with idiopathic dystonia. All patients were implanted with two SenSight (Medtronic) DBS electrode (left and right GPi), which include eight platinum–iridium contacts of 1.5 mm each in a 1-3-3-1 configuration and a contact-to-contact separation of 0.5 mm. This lead was specifically designed for directional sensing to minimize signal interference and recently got the FDA and CE mark approval for clinical use [10]. Before starting the LFP recordings, an impedance test is always performed automatically to exclude lead encapsulation-related problems. In case of potential short (<250 ohms) or open (>10 Kohms) circuits, the sense channels involved are excluded. Surgery was performed using the Vantage frame (Elekta, Stockholm, Sweden) under general anesthesia with immediate verification of lead location with intra-operative CT scan (O-Arm, Medtronic). One month after surgery, all patients underwent brain MRI to rule out surgical complications and to verify correct lead placement.

### 2.2. LFP/MEG Acquisition Protocol

The MEG/LFP acquisition protocol was conducted the day before DBS surgery (T0), 1 month (T1), and 3 months (T2) after implantation. At T0 MEG signal was recorded at the baseline without any implanted device and under resting conditions for 3 min with eyes closed and 3 min with eyes open. At T1 and T2 the protocol consisted of 2 MEG acquisitions, each conducted for 3 min with eyes closed and 3 min with eyes open: (1) resting state without LFP recording and (2) resting state with LFP recording. Chronic GPi stimulation was started after T1.

- LFP signal acquisition

LFP signals were acquired through the DBS device with two different modalities: Indefinite Streaming (IS) and BrainSense (BS). The IS mode allows continuous acquisition and recording of LFPs from three electrode pairs per hemisphere, with a sampling rate of 250 Hz and an FFT resolution of 0.98 Hz. The signal is filtered by a low-pass filter at 100 Hz and a high-pass filter at 1 Hz, with an additional adjustable filter at either 1 or 10 Hz. This mode represents a significant advancement over prior DBS systems, which were limited to episodic or short-duration LFP recordings. The BS mode allows recording the LFP signal at the same sampling rate (250 Hz) and FFT resolution (0.98 Hz) as the IS mode, while also delivering stimulation concurrently.

- MEG signal acquisition

A 306-channel whole head MEG system (Triux, MEGIN, Helsinki, Finland) was used to collect the MEG signals. Pairs of electrodes positioned bilaterally 2–3 cm apart over the belly of the right and left flexor and extensor of the wrist were used to simultaneously record surface EMG signals. Moreover, bipolar electro-oculographic (EOG) and electrocardiographic signals (ECG) were acquired. All signals were sampled at 1 kHz. A 3D digitizer (FASTRAK, Polhemus, Colchester, VT, USA) was used to digitally capture the locations of five coils on the participant’s scalp, three anatomical landmarks (the nasion, right and left preauriculars), and additional scalp points before the recording for the purpose to continuously monitor the participant’s head position inside the MEG helmet and to co-register MEG signal and MR images. To characterize instrument and environmental noise, 2 min of empty room recordings were collected just before the patients’ acquisition. To remove external interference and correct for head motions, the raw MEG data were first pre-processed off-line using the spatio-temporal signal-space separation approach [11] implemented in the Maxfilter 2.2 (MEGIN, Helsinki, Finland). The data were then band-pass filtered at 0.1–100 Hz. Cardiac and ocular movement artifacts were removed using the ICA algorithm based on EEGLAB toolbox [12] implemented in a custom-made MATLAB code (R2021a, Mathworks Inc., Natick, MA, USA), using ECG and EOG as reference. To assess potential degradation of signal quality, we evaluated the Signal-to-Noise Ratio (SNR) across the 1–30 frequency range as the ratio of the signal power of a 2 min MEG recording of the patient in a resting state with eyes closed to the signal power of the recording in the empty room. All the analyses were performed using Brainstorm software and custom-made Matlab-based scripts.

- MEG-LFP synchronization method

The two main challenges in simultaneous MEG-LFP acquisition are potential artifacts on MEG signals due to the presence of a stimulator and the difficulty of offline signal synchronization. For the first issue, proper positioning of DBS transmitter and receiver was adopted. The communicator was placed as far as possible from the MEG sensor array, while the recording tablet was placed outside the magnetically shielded room and connected to the communicator via a cable passing through a shielded hole.

**Figure 1:**
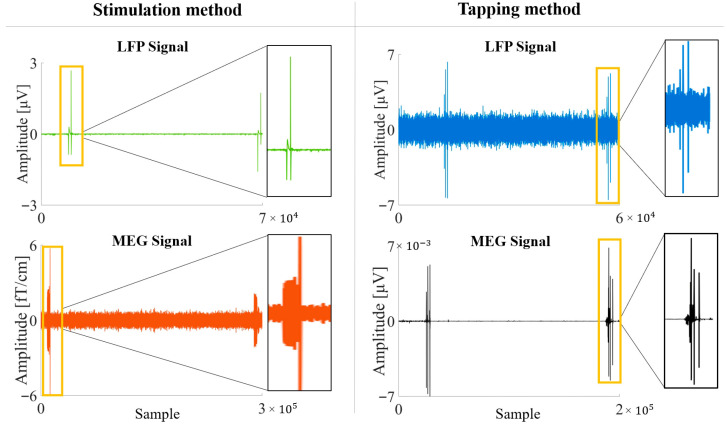
High-quality signal from Subject 5. Yellow rectangles highlight the artifacts used to align the LFP-MEG signals and to define epochs.

To obtain synchronizable recordings, two methods were adopted. In the first one (Tapping method), the stimulator was set to IS mode. At the beginning and end of each acquisition, an operator physically tapped three times on the Implantable Pulse Generator (IPG) site (chest or abdomen), where an accelerometer had been positioned. The tapping sequence consisted of a one-second interval between the first and second taps, and a two-second interval between the second and third taps. The mechanical impulse is transmitted from the IPG along the entire length of the implanted hardware, creating mechanically introduced noise. This results in a distinct artifact on the LFP signal, which appears simultaneously on the accelerometer trace recorded by the MEG equipment (Figure 1).

In the second method (Stimulation method) the stimulator was set to BS mode and, at the beginning and at the end of each acquisition, a sham stimulation (0.2 mA at 125 Hz for 90 μs) was delivered by the stimulator, reproducing an artifact on the MEG signal (Figure 1).

To detect and mark artifacts from tapping and stimulation, a consensus-based approach was employed to minimize subjectivity. Two experienced MEG operators (EV and DD) and two experienced LFP operators (VL and LB) inspected the respective signals simultaneously. Instead of independent scoring, the operators worked together to reach full agreement on both the identification of the artifacts and their precise temporal landmarks (specifically, the offset of the first artifact and the onset of the second). Afterward, the signals were downsampled to the LFP sampling frequency; finally, MEG and LFP signals were aligned.

### 2.3. Data Analysis

We collected the following parameters for the subsequent analysis: number of patients with detectable artefacts, and the phase shift error for both the Tapping and the Stimulation method. Moreover, we compared the SNR between the post-implantation and pre-implantation conditions for both MEG gradiometers and magnetometers (Wilcox signed-rank test; *p* < 0.05 was considered significant).

## 3. Results

### 3.1. MEG Signal Quality

**Figure 2:**
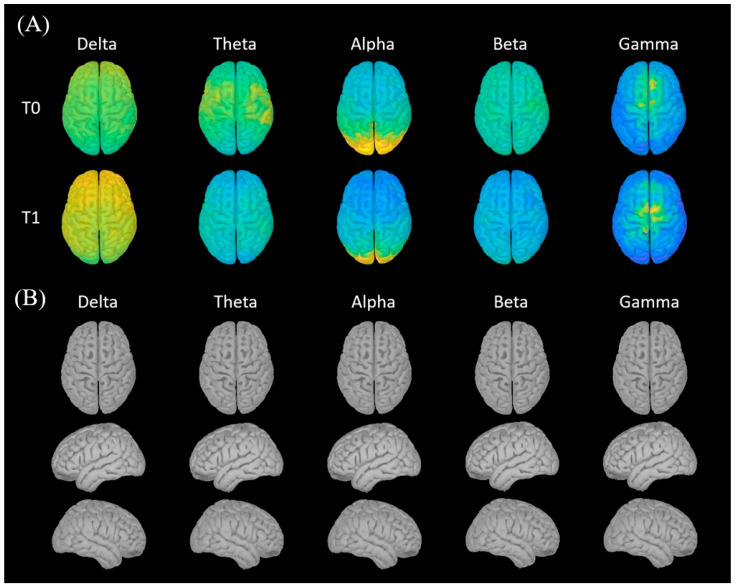
Resting-state MEG source reconstruction and statistical comparison between pre- (T0) and post-implantation (T1). Source-reconstructed cortical activity is shown across five canonical frequency bands (delta: 1–4 Hz; theta: 4–8 Hz; alpha: 8–12 Hz; beta: 13–30 Hz; low gamma: 30–45 Hz). (**A**) Grand-average source maps across the entire cohort (N = 11). Note that measurement scales differ across frequency bands but are fixed between T0 and T1 to facilitate comparison. (**B**) Group-level within-subject comparison (T0 vs. T1) across the same frequency bands. Statistical significance was assessed using a non-parametric, cluster-based permutation test with FDR correction for multiple comparison. No significant differences were found.

The comparison of the SNR between the post-implantation and pre-implantation conditions revealed a mean difference of −0.12 ± 1.85 dB on gradiometers and of −0.47 ± 2.03 dB on magnetometers. No significant difference was found in SNR between pre- and post-implantation conditions (gradiometers: Z = −0.663, *p* = 0.508, r = 0.20; magnetometers: Z = 1.244, *p* = 0.214, r = 0.37). The median SNR change was –0.12 dB (95% CI: −1.8 to +1.3 dB) for gradiometers and –0.47 dB (95% CI: −2.0 to +1.2 dB) for magnetometers, indicating that the observed variations were small and not practically significant. To further verify that the presence of the DBS hardware did not distort the spatial topography of neuromagnetic activity, we performed source reconstruction across canonical frequency bands. As shown in Figure 2, the cortical activity patterns remained stable between the pre-implantation (T0) and post-implantation (T1) sessions, confirming the preservation of signal topography.

### 3.2. Stimulation Method

**Figure 3:**
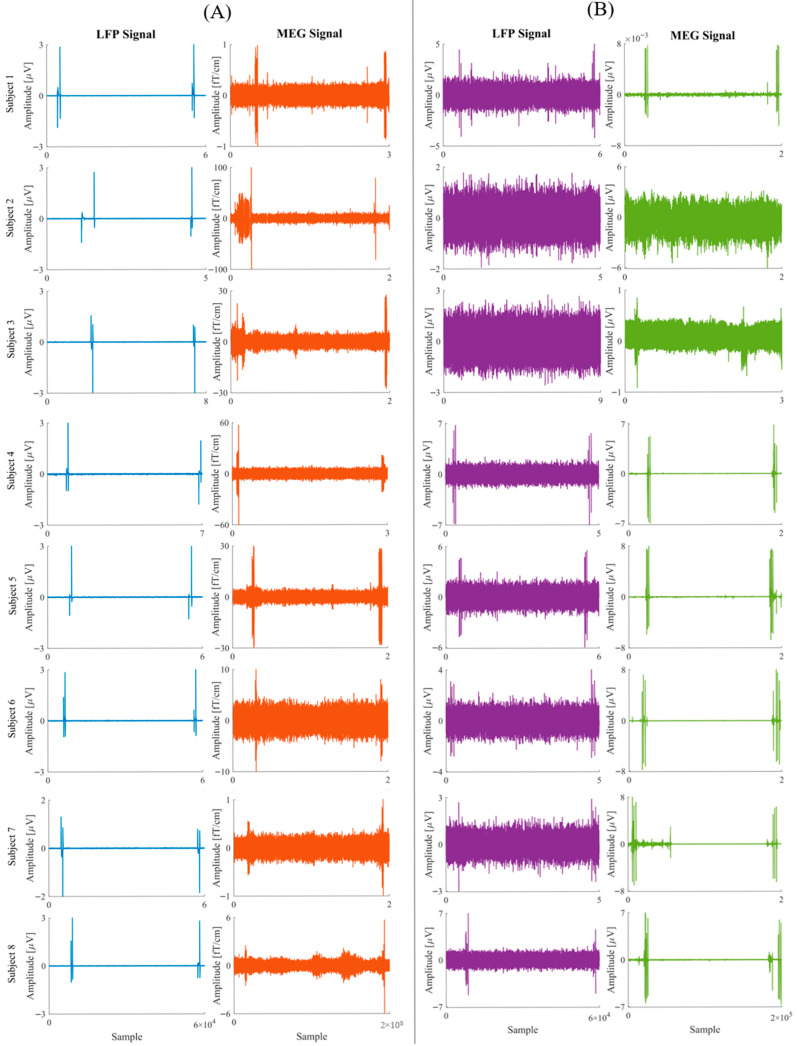
LFP and MEG recordings from all patients for whom data were analyzable in both Stimulation method (left panel—(**A**)) and Tapping method (right panel—(**B**)). The subject numbers in the figure are indexed based on the analyzable dataset; for instance, “Subject 1” refers to the fourth patient in the study. To improve clarity, common axes are shown only on the first or last sub-image of each panel.

In 2 patients (18.2%), LFP data were corrupted and not usable for analysis. With the stimulator method, artifacts were clearly identifiable in both LFP and MEG signals in all patients (100%) with analyzable dataset (Table 1—Figure 3A). The artifacts generated by the stimulator were highly consistent, with clear temporal markers indicating the beginning and end of the stimulation periods. Since the artifact is generated by a repetitive stimulation pattern with a well-defined frequency, it was easily distinguishable from the underlying brain activity, allowing precise temporal alignment of the two signals.

### 3.3. Tapping Method

In 3 out of 11 patients (27.3%), LFP data were corrupted and not usable for analysis. In the remaining 8 patients (72.7%), the tapping artifact was identifiable in the LFP signal in 6 patients (75.0%) on at least one contact. Similarly, the tapping artifact was clearly detectable on both MEG signal and accelerometer in 6 out of 8 patients (75.0%). Both the LFP and MEG artifacts were undetectable in the same 2 patients (25.0%) (Figure 3B—Table 1). Artifact peaks were identified on signals as the third peak of the first tapping sequence (beginning of acquisition) and the first peak of the second tapping sequence (end of acquisition).

### 3.4. LFP Data Integrity

**Figure 4:**
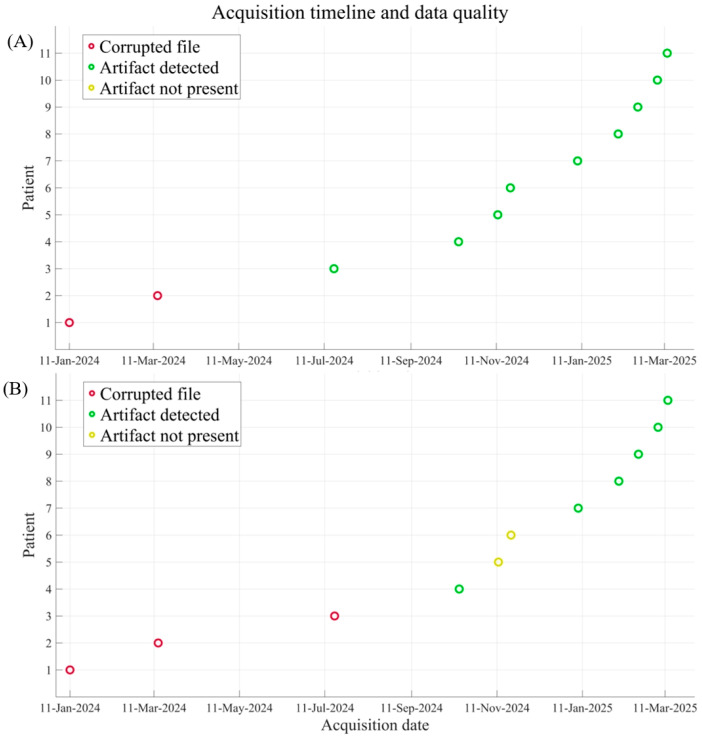
Each circle represents a single data acquisition of LFP signals. MEG recordings showed the same trend and are not reported for simplicity. Data are grouped by acquisition modality: Stimulation (panel (**A**)) and Tapping (panel (**B**)). The temporal distribution highlights the evolution of data quality over time, with a tendency toward improved signal quality in later sessions.

The corrupted LFP data were due to a technical issue related to the data acquisition and transfer process: intermittent, brief dropouts in the communication link between the DBS transmitter, located inside the MEG shielded room, and a receiver tablet outside, were not detected in real time, resulting in an incomplete data stream. However, these technical challenges were primarily confined to the early phase of the study. As the study progressed, we observed a clear improvement in system stability, mirroring the fact that the execution and consequent detection of ‘Tapping’ artifacts became more consistent and identifiable over time, as evidenced in Figure 4.

### 3.5. MEG-LFP Alignment

The alignment procedures resulted in a phase shift error of 91 ± 22 ms and 288 ± 166 ms, for the tapping and stimulation method, respectively (Table 1).

## 4. Discussion

This study demonstrated the feasibility of achieving simultaneous MEG and LFP acquisition with excellent results in terms of cortical signal quality, enabling the evaluation of complex brain dynamics in a more comprehensive way. Our results are consistent with those previously reported by Oswal et al. [9], Werner et al. [13], and Berki et al. [14] who emphasized the complementary nature of MEG and LFP in studying brain oscillations relevant to movement disorders.

The practical issues addressed in this study concerned both the possibility of obtaining high-quality signals from both methods and the possibility of aligning the two signals. Acquiring the LFP signal required only a few adjustments to the position of the transmitter so that the signal could be picked up outside the magnetically shielded room. The small effect sizes observed for the SNR comparison further support the conclusion that the implantation procedure does not substantially affect MEG signal quality. The minimal change in both gradiometers and magnetometers confirms the robustness of the acquisition protocol and the feasibility of simultaneous MEG–LFP recordings without relevant degradation of cortical signal. The more pronounced decrease in SNR for magnetometers compared to gradiometers is expected, as magnetometers are generally more sensitive to environmental noise and artifacts. The lack of significant variation across individual sensors indicates that the implantation had a uniform effect on signal quality across the entire MEG system, suggesting that the implantation process does not substantially interfere with the overall MEG recordings.

The simultaneous recording of MEG and LFP overcomes the intrinsic limitations of using either technique in isolation. As noted, MEG captures the magnetic correlates of cortical pyramidal activity with high temporal resolution and minimal distortion from skull conductivity (“magnetic transparency”). However, its sensitivity decreases with depth, making the precise resolution of deep subcortical sources challenging. Conversely, LFPs provide a direct, high-amplitude electrical readout of local neuronal populations within the GPi, but lack spatial coverage of the cortex. By combining these modalities, our setup effectively bridges the macroscopic and microscopic scales. This integration fits within a broader context of recent studies, such as those by Litvak et al. [15] and Wehmeyer et al. [16], which highlight how functional connectivity analyses of the cortico-basal ganglia-thalamo-cortical networks can advance our understanding of circuit topology. This is particularly relevant for dystonia, which is increasingly conceptualized not as a focal lesion, but as a network disorder involving dysfunctional oscillations within these loops.

Regarding the LFP acquisition, we assessed two methods for LFP recording: Intra-operative (IS) and BrainSense (BS). The BS method is limited to acquiring signals from a single pair of contacts per hemisphere but offers the distinct advantage of allowing stimulation during recording, which aids synchronization with MEG. On the other hand, IS method grants the possibility to access all available contacts on the implanted electrodes, but it does not permit simultaneous stimulation. The flexibility in LFP recording approaches we observed is similar to the findings of Hnazaee et al. [8], who successfully combined MEG with telemetric intracranial recordings, highlighting the practical trade-offs between spatial resolution and stimulation compatibility. Globally, both methods can be used, with dedicated adjustments.

The alignment of the LFP and MEG signals was achieved with two methods, Tapping and Stimulation. Stimulation artifacts are highly detectable due to their consistent and repetitive nature. They can be easily identified in MEG recordings as unusually high frequency activity. This method offers precision, but the number of available LFP recordings is limited by the method itself. Moreover, attention should be paid to artifacts due to harmonics and composition with the physiological signal; therefore, it is recommended that periods close to stimulation should not be selected as the beginning and end of the epoch to be analyzed. Tapping on the DBS device provides flexibility but requires careful standardization of both execution and detection. Tapping artifacts are less consistent and may vary in detectability depending on the force and location of the tap. However, this method enables acquisition from all available LFP contacts and has a lower impact on signal characteristics. Acquiring signals in both modalities is advantageous for gathering more information, and it likely represents the best operative solution. The demonstrated synchronization methods with tolerable temporal error support reliable frequency domain analyses, echoing artifact management strategies recommended by Oswal et al. [9] that emphasize careful epoch selection and visual inspection for artifact handling.

Synchronization accuracy and precision showed a clear improvement over the course of the study. This trend, evidenced by reduction in temporal variability and more consistent artifact identification in later sessions, likely reflects increased operator familiarity with the acquisition protocol and refinements in the technical setup. Both methods ultimately demonstrated the potential to synchronize recordings with acceptable temporal error for integrated analyses. Specifically, frequency-domain techniques such as power spectral density estimation, which rely on averaging over multiple epochs, can tolerate minor timing discrepancies. These methods are designed to capture consistent patterns across frequencies, making them robust to slight misalignments—a strategy echoing artifact management recommendations by Oswal et al. [9].

This study presents some limitations. First, the sample size is relatively small, although patients with dystonia eligible for DBS are quite rare. Second, artifact identification relied on visual inspection by expert raters, which may introduce a degree of subjectivity. Third, the lack of a shared hardware trigger between MEG and DBS systems introduces non-negligible temporal variability, particularly in the Stimulation method. This delay arises from a combination of technical factors, including intrinsic jitter in the DBS switching mechanism and the difficulty of visually isolating the exact onset of stimulation artifacts when superimposed on ongoing physiological MEG activity. Consequently, while current synchronization accuracy is sufficient for frequency-domain analyses, it limits the feasibility of strict time-domain analyses requiring millisecond-level precision. To address this, future work will focus on implementing automated artifact detection algorithms and advanced signal processing techniques to minimize this temporal offset.

Despite these limitations, our results align with prior studies linking subcortical oscillatory biomarkers to motor symptoms and DBS efficacy in movement disorders (Neumann et al. [4]; Lofredi et al. [5]). This integration strengthens the clinical and mechanistic relevance of simultaneous MEG-LFP studies for advancing neuromodulation therapies (Asadi et al. [17]; Horn & Fox [18]). Our future studies will analyse the two signals asynchronously, to characterise temporal dynamics in LFP and cortical activity, and synchronously, to elucidate potential functional relationships between them. These results pave the way for integrated studies of the interactions between the cortical and subcortical structures involved in the extended circuitry underlying dystonia, allowing the verification of the plastic modifications of the structure over time due to GPi stimulation.

## 5. Conclusions

This study evaluates the technical feasibility and reliability of simultaneous whole-head MEG and intracranial LFP recordings in patients with dystonia treated with Deep Brain Stimulation. We demonstrated that the presence of the implanted DBS hardware does not significantly compromise the quality or topographical integrity of neuromagnetic recordings, preserving the ability to perform accurate cortical source reconstruction.

We validated two complementary synchronization strategies, identifying a clear operational trade-off: the Tapping method maximizes spatial coverage by enabling access to all electrode contacts, whereas the Stimulation method offers superior consistency despite limited channel availability. This adaptable methodological frame-work effectively bridges the gap between macroscopic cortical dynamics and microscopic subcortical activity, overcoming the intrinsic limitations of single-modality recordings.

By providing a comprehensive view of the cortico-basal ganglia-thalamo-cortical loop, this multimodal approach provides an innovative framework to investigate the complex network dysfunctions underlying dystonia.

## Figures and Tables

**Table 1:** Clinical data and epochs measures for all patients. Pat.—Patient; PKAN—Pantothenate kinase-associated neurodegeneration; DYT28—Dystonia 28, childhood-onset dystonia; DYT6—Dystonia 6, adolescent-onset dystonia of mixed type; DYT11—Dystonia 11, myoclonus dystonia; DYT12—Dystonia 12, rapid-onset dystonia parkinsonism. Data marked with an asterisk (*) refers to trials in which artifact identification and, consequently, accurate epoch selection were particularly challenging according to both operators. N/A: missing data due to technical issues.

Pat.	Sex, Age (Years)	Diagnosis	DBS Side	Stimulation Method MEG-LFP Difference [s]	Tapping Method MEG-LFP Difference [s]
**1**	**F, 18**	PKAN	Right	N/A	N/A
**2**	**M, 14**	DYT28	Right	N/A	N/A
**3**	**F, 46**	DYT6	Right	0.615	N/A
**4**	**M, 21**	Idiopathic dystonia	Left	0.082	0.135
**5**	**M, 11**	DYT28	Right	0.183	*
**6**	**M, 14**	PKAN	Right	0.246	*
**7**	**M, 46**	Idiopathic dystonia	Right	0.219	0.073
**8**	**M, 17**	Idiopathic dystonia	Right	0.331	0.089
**9**	**M, 14**	DYT11	Right	0.228	0.086
**10**	**M, 48**	DYT12	Right	0.204	0.079
**11**	**M, 47**	Idiopathic dystonia	Right	0.032	0.085
				**0.288 ± 0.166**	**0.091 ± 0.022**

## Data Availability

Raw data were generated at IRCCS Fondazione Istituto Neurologico Carlo Besta. Derived data supporting the findings of this study are available from the corresponding author [VL] on request.

## Abbreviations

The following abbreviations are used in this manuscript:

LFP	Local field potential
MEG	Magnetoencephalography
GPi	Globus pallidus internus
DBS	Deep brain stimulation
IS	Indefinite Streaming
BS	Brain Sense
FFT	Fast Fourier transform
EOG	Electro-oculographic
ECG	Electro-cardiographic
ICA	Independent component analysis
SNR	Signal-to-Noise ratio
